# The Stockport Infirmary

**Published:** 1894-02-24

**Authors:** 


### THE STOCKPORT INFIRMARY.

The outcome of a small dispensary started by the benevolence of Dr. Briscoll in 1790 is the Stockport Infirmary of to-day. Medicines were dispensed to the necessitous poor in a small cottage near Wellington Bridge for the first time just a little over one hundred years ago. One wonders that so ancient a centre as Stockport, or Stopford, as it was known in the days of the Romans, should not have had some provision for the sick poor long before Dr. Briscoll's time. Stockport, however, has made up for lost time in later years, and the infirmary to day is a very creditable institution, and a testimony to the philanthropy of its prominent citizens. Facing Wellington Road, with a small grass plat in front, the infirmary is of the architecture of the period of the later Georges, a long two-storey building with a heavy Greco stone portico in the centre. The windows in front light the rooms in the main building, which are occupied by the staff. These rooms open on to two long corridors, from which abut three wings containing the wards. The wings, which are known as pavilions, are, in virtue of that excellent system, well lighted and ventilated, and cheerful in aspect. One pavilion is occupied by women, one by children, and the other by the men. Two of the larger wards are heated by double stoves in the centre of the room, which I think is probably a better way of distributing the heat than the ordinary fashion of the stoves at either end of the ward. The Nurses and Sisters, however, do not seem to care for the fires in the centre of the wards; the pipes and stove block a general view of the beds and their occupants, and certain restless cases require the nurse to be always on the watch.

The centre pavilion has only one small ward in the upper storey. The rest of the space is taken up by the operation room. On the lower storey of this wing are the accident ward and dispensary. At the back of the pavilion are the laundry (which is admirable in its appointment) and a new disinfecting stove and chamber after the latest and most approved principles. The only drawback in the infirmary is the kitchen, which is in the central wing, and dispenses the odours of its savoury and unsavoury dishes all over the building. This trouble was only too palpable as the surgeon deputed to escort me received me at the door, and the conglomerate atmosphere of stewing meats and vegetables followed us everywhere. There are seven large wards in this infirmary, containing twelve beds each, and there are two small rooms for isolated cases. The patients who are able to move about the wards wear scarlet woollen jackets, which add to the brightness of the place. The children's ward was full of little patients. In fact the accommodation is quite inadequate to the demand. A hospital for children is absolutely a necessity to-day in Stockport. The surgeons are obliged to treat the most urgent cases which cannot be admitted to the infirmary in the little patients' homes. The difficulties of proper sanitary precautions and nursing under these conditions are only too obvious. The present infirmary, if the directors had the means and intention, could not very well be extended. The ground over which there was any possibility of the hospital being enlarged was sold by the directorate about thirty years ago to a railway company. There is much to be desired in the accommodation of the nurses in this hospital; two and sometimes three are obliged to sleep in one room.

